# Growth and stress response in *Arabidopsis thaliana*, *Nicotiana benthamiana*, *Glycine max*, *Solanum tuberosum* and *Brassica napus* cultivated under polychromatic LEDs

**DOI:** 10.1186/s13007-015-0076-4

**Published:** 2015-04-30

**Authors:** Martin Janda, Oldřich Navrátil, Daniel Haisel, Barbora Jindřichová, Jan Fousek, Lenka Burketová, Noemi Čeřovská, Tomáš Moravec

**Affiliations:** Laboratory of Virology, Institute of Experimental Botany AS CR, Rozvojová 313, 165 02 Prague 6, Czech Republic; Laboratory of Pathological Plant Physiology, Institute of Experimental Botany AS CR, Rozvojová 313, 165 02 Prague 6, Czech Republic; Laboratory of Stress Physiology, Institute of Experimental Botany AS CR, Rozvojová 313, 165 02 Prague 6, Czech Republic; Department of Biochemistry and Microbiology, University of Chemistry and Technology Prague, Technická 5, 166 28 Prague 6, Czech Republic

**Keywords:** LED, Fluorescent tubes, Plant physiology, Light, *Arabidopsis thaliana*, *Nicotiana bentamiana*, Potato, Soybean, Oilseed rape

## Abstract

**Background:**

The use of light emitting diodes (LEDs) brings several key advantages over existing illumination technologies for indoor plant cultivation. Among these are that LEDs have predicted lifetimes from 50–100.000 hours without significant drops in efficiency and energy consumption is much lower compared to traditional fluorescent tubes. Recent advances allow LEDs to be used with customized wavelengths for plant growth. However, most of these LED growth systems use mixtures of chips emitting in several narrow wavelengths and frequently they are not compatible with existing infrastructures. This study tested the growth of five different plant species under phosphor coated LED-chips fitted into a tube with a standard G13 base that provide continuous visible light illumination with enhanced blue and red light.

**Results:**

The LED system was characterized and compared with standard fluorescence tubes in the same cultivation room. Significant differences in heat generation between LEDs and fluorescent tubes were clearly demonstrated. Also, LED lights allowed for better control and stability of preset conditions. Physiological properties such as growth characteristics, biomass, and chlorophyll content were measured and the responses to pathogen assessed for five plant species (both the model plants *Arabidopsis thaliana*, *Nicotiana bentamiana* and crop species potato, oilseed rape and soybean) under the different illumination sources.

**Conclusions:**

We showed that polychromatic LEDs provide light of sufficient quality and intensity for plant growth using less than 40% of the electricity required by the standard fluorescent lighting under test. The tested type of LED installation provides a simple upgrade pathway for existing infrastructure for indoor plant growth. Interestingly, individual plant species responded differently to the LED lights so it would be reasonable to test their utility to any particular application.

**Electronic supplementary material:**

The online version of this article (doi:10.1186/s13007-015-0076-4) contains supplementary material, which is available to authorized users.

## Background

The use of light emitting diodes (LEDs) brings several key advantages over existing illumination technologies for indoor plant cultivation. Both fluorescent tubes and high pressure sodium lamps generate a lot of heat that must be removed from closed environments such as growth rooms and growth chambers, creating additional issues with the control of air-flow, humidity and irrigation. Dealing with all these processes contributes to the energy consumption of the whole system. LEDs also offer very long predicted lifetimes in the range of 50–100.000 hours without significant drops in efficiency and thus do not need to be periodically checked and replaced. LEDs allow simple control of the light intensity and in some settings also of its spectral composition. Most LEDs operate on low voltage direct current (DC), which may offer additional safety benefits in a humid environment with splashing water such as growth chambers. Besides their advantages in energy-efficiency, LED-based light sources also generally have a good safety profile: they do not contain fragile glass or mercury and other hazardous chemicals, and they can be safely touched without gloves during operation. Fluorescent lamps, which are currently the most common source of light for indoor cultivation emit light in several discrete wavelengths ranging from 350 to 750 nm which are not always aligned with the wavelengths absorbed by a plant’s photosynthetic apparatus and thus inevitably generates unnecessary heat. Most fluorescent tubes emit light in all directions (360°) and thus much of the light is not efficiently used by the plants. Based on the known advantages of LEDs, scientists had immediately started to think about their possible use in horticultural lighting [1-3].

The use of LEDs for plant growth was first suggested by Bula et al. (1991) [4]. They studied lettuce plants under red LEDs supplemented by blue fluorescence lamps. At the time red LEDs were the most efficient and they emit light that corresponds to the absorbance peak of chlorophyll (660 nm). However it was known that blue light is also important for plant development and morphology [3,5-9] yet blue LEDs were then unavailable.

In early attempts in their use LEDs were only available in certain colors (red being the most common) and the intensity of emitted light was low. Also the price of LEDs made their use prohibitive for most applications except for experiments with plant growth during space missions [10-12]. Since those times continued improvements in LED technology, along with an exponential decline in their cost, have made them an attractive choice for many applications including that of indoor plant growth systems.

Today LED technology is well established among manufacturers of light sources designed specifically for plant growth (e.g. Philips GreenPower LED product line). However most commercial LED light sources use narrow band LED chips specifically mixed for the purpose of plant growth [13] and usually require existing growth systems to be refitted both electrically and mechanically. In this study we have used polychromatic continuous spectrum LED chips which were fitted into a standard G13 light fitting that, already contained all the electronics necessary to convert 220 AC current to low voltage DC. No additional ballast is required. While multiple manufacturers provide LED tubes for direct replacements of fluorescent tubes (e.g. Valoya L series, Philips CorePro, Osram SubstiTube and others) however the light output of most of these solutions is not specifically designed to match requirements of plants. In our previous experiments we have achieved poor growth of some plant species (*N. benthamiana)* under LED tubes providing both warm-white and cool-white illumination, thus for this set of experiments we selected tubes, which have enhanced emission in blue and especially in red part of visible spectrum. Since the application of LEDs for indoor plant cultivation is very attractive field, we expect that similar plant-oriented LED tubes are or soon will be offered by multiple manufacturers and thus the results described in this report might be of interest to the community.

In this report we have selected several model plant species which are widely used by the plant research community. The group included: *Arabidopsis thaliana,* the most important model organism used in plant biology and genetics as well as in the study of plant-pathogen interactions; *Nicotiana benthamiana,* a popular model species in plant virology and the study of RNA silencing; soybean (*Glycine max*, cv Jack), the most important legume crop and which is also used in our laboratory to study its potential to express and accumulate pharmaceutically valuable proteins such as vaccines and antibodies in its seeds [14,15]; potato (*Solanum tuberosum*, cv. Kamýk), an important food crop which is used in our laboratory to study sugar metabolism and virus resistance [16-18]; and oilseed rape (*Brassica napus*, cv Columbus), an important oilseed crop which is used in our lab in studies of plant-pathogen interactions [19,20]. In our study we demonstrated that LED tubes provide a viable alternative to current fluorescent tubes. Most of the tested plant species showed only minor differences in their growth rate and physiology; however, LEDs emitted much less heat and thus simplified the control of temperature and humidity. While the initial investment to replace the fluorescent tubes with LEDs is substantial, their use is economical in the long run.

## Results

### Light quality and intensity

**Figure 1:**
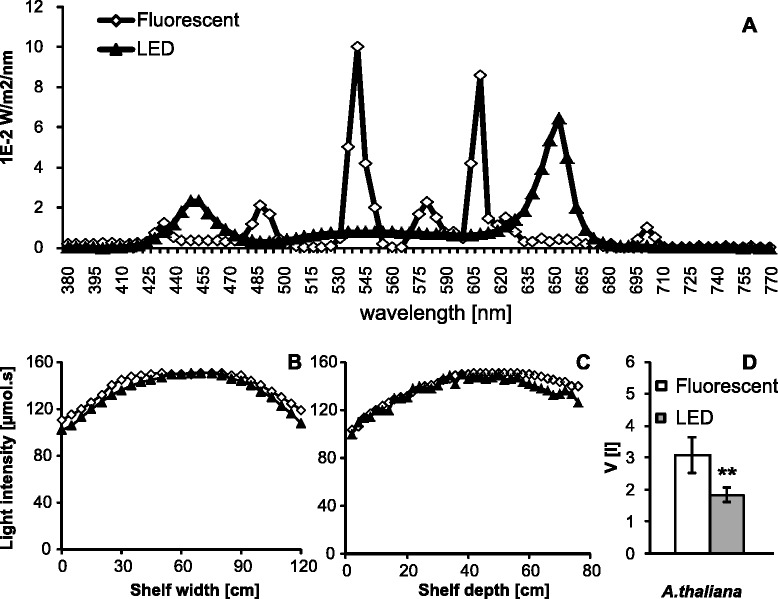
Conditions characteristics **A)** Spectral composition of visible light produced by the illumination sources used in this study. The graph is based on data provided by manufacturers and normalized to the same total visible light output. **B)** and **C)** show PAR intensity measured in both axes of the shelf at a distance of 40 cm from the light source. Data points from one of the three technical replicates are shown. The datapoints represent the average from three measurements. Standard deviations were within 2% of measured values. **D)** Total water consumption after one month of the different plant species. *A. thaliana* tray contains 24 pots/plants with jiffy tablets. It was made in three biological replicates. Error bars represent SD. Asterisks indicate statistically significant differences compared to plants grown under fluorescent lights (** P < 0.01, two tailed Student’s *t*-test).

The spectral characteristics of both fluorescent and LED lights are depicted in Figure 1A. Fluorescent tubes emit light of several narrow bands, the prominent being 405 nm, 435 nm, 490 nm, 545 nm, 585 nm, 615 nm and 710 nm, while the GrowLED lights provide a full continuous spectrum with enhanced emission peaks around 445 nm and 660 nm. The photon flux density (PFD) of fluorescent tubes had a blue:green:red ratio (defined as 400–500 nm for blue, 501–599 nm for green and 600–700 for red, see also [21]) with a ratio of 16.1: 45.4: 38.5 while the same ratio for the LED tubes was 19.1:19.8:61.1. The LED tubes thus emitted a much higher proportion of red light and substantially less green light, while the amount of blue wavelengths was similar for both sources. Interestingly, the LEDs provided only very little of far-red light, the red:far-red ratio being only 61:1, while the same ratio for fluorescent lamps was 8.5:1.

Photon flux density (PFD) was measured using Li-COR Quantum Photometer LI-185B (see methodology section) in a dense matrix (6x13x3 measurements) over the whole area of the shelf at three different distances from the light source. In Figure 1B and C we show the resulting light density profiles at the base of the shelf (h = 0 cm, i.e. 43 cm from the light source) across the shelf width and depth. Both light sources provided similar PFD intensities; however, the light field from fluorescent tubes was more uniform.

### Temperature

The important advantage of solid state LEDs over fluorescent tubes would have to be lower heat emissions. We therefore measured the temperature in a matrix of 3x3 points over the shelf area using mercury thermometers. Temperature was also recorded over the 24-hour period at 5 minute intervals using a USB datalogger (Table 1). It is apparent that while the temperature in the growth chamber was efficiently controlled within a close interval above the set temperature (22 +/− 0.5°C), the temperatures on the shelf and between the plants form more complex pattern. Especially in the case of fluorescent tubes we have identified very strong temperature gradients. When the plants were properly irrigated the temperature measured at the rosette of Arabidopsis leaves was 24.5 +/− 0.2°C in shade and 27.0 +/− 0.8°C in non-shade conditions, however only several centimeters from growing plants at the same height over the shelf and between the water filled trays reached 30.3 +/− 0.3°C in shade and 31.3 +/− 0.8°C in non-shade conditions. In the case of LED tubes the gradient between open shelf area and individual plants was much milder 23.0 +/− 0.3°C on Arabidopsis leaves in shade and 24.8 +/− 0.2 in noon-shade conditions. Without the Arabidopsis the temperature was 25.0 +/− 0.5 in shade and 27.0 +/− 0.5°C in non-shade conditions (Table 1). It is clear that the heat generated by the fluorescent tubes could be an important factor contributing to differences in the studied plant physiology. The differences in temperatures were reflected also by the differing water requirements. Trays under LED lights required around 40% less water than trays under fluorescent lights (Figure 1D). We have also measured the electric power consumption of whole shelves equipped either by LED diodes or fluorescent tubes during the course of the experiment using a SOLID Digital electricity meter. The average power consumption of fluorescent tubes was 42 W per tube (including starter and ballast), whereas that of LEDs was 16.3 W per tube.

**Table 1:** **Temperatures under distinct conditions**

		**Fluorescent**		**LED**	
		**Mean (°C)**	**+/− (°C)**	**Mean (°C)**	**+/− (°C)**
**Non-Arabidopsis	Shaded	30.3	0.3	25.0	0.5
	Non-shaded	31.3	0.8	27.0	0.5
*Arabidopsis	Shaded	24.5	0.2	23.0	0.3
	Non-shaded	27.0	1.0	24.8	0.2

Temperatures were measured 7 cm above the shelf *either on Arabidopsis leaves (Arabidopsis) **or on the pot without Arabidopsis plants (non-Arabidopsis). The datalogger was shaded or non-shaded by the alluminium cover.

### Response of individual plant species

#### Arabidopsis thaliana

**Figure 2:**
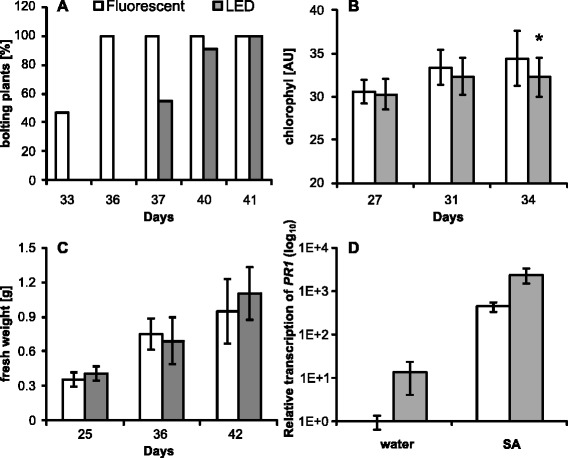
Growth of *A. thaliana* plants under different illumination sources. **A)** Bolting age of Arabidopis plants. n = 15 (fluorescent), n = 11 (LED). **B)** Measurement of chlorophyl content at different time points. n = 15 plants (mean from 3 leaves from one plant). **C)** Fresh weight at different time points. n = 11 (25; 42 days), n = 6 (36 days). **D)** Relative expression of *PR-1* gene in 5 weeks old plants after treatment with 300 μM NaSA. Values represent 2 independent samples from 2 biological replicates. The *PR-1* expression was normalized to reference gene *SAND*. In all cases error bars represent SD. Asterisks indicate statistically significant differences compared to plants grown under fluorescent lights (*P < 0.05; two tailed Student’s *t*-test).

*Arabidopsis thaliana* is the most commonly used model plant species in plant science worldwide. In this study we have compared several physiological parameters of *A. thaliana* plants grown under LED illumination with plants grown under standard fluorescent tubes. We measured the fresh weight and dry weight of whole rosettes of Arabidopsis plants (Figure 2C and Additional file: 1 Figure S2B). Plants of three different ages (25, 35, 42 days) were weighed. The fresh weight was similar in both groups in all age categories (Figure 2C). In older plants (42 days) the dry weight was higher in plants grown under the LED lights (Additional file: 1 Figure S2B). Further, we have measured the chlorophyll content of plants 27, 31 and 34 days old (Figure 2B). The chlorophyll content was generally similar with the exception of older (34 days) plants where the LED grown plants showed a non-significant decrease of chlorophyll compared to plants grown under fluorescent tubes (Figure 2B). The most pronounced difference was a delayed start of bolting under LED lights (Figure 2A; Additional file: 1 Figure S2D). Also, the LED grown plants become purple faster after 7 weeks (Additional file: 1: Figure S2C).

An object of study of our laboratory are plant-pathogen interactions [19,20,22], thus we were also interested in the impact of illumination on a plant’s response to treatment with the important defense phytohormone salicylic acid (SA). We measured the transcription of the *PR1* (*PATHOGENESIS RELATED* 1) gene which is a marker gene of the SA signaling pathway [23]. The only difference between plants grown under the different light sources was a non-significantly elevated basal transcription of the *PR1* gene (without treatment – control plants; Figure 2D) under LED illumination. Eventually we tested the response of *Arabidopsis thaliana* to the commonly-used pathogenic bacteria *Pseudomonas syringae* pv *maculicola* ES4326. Bacterial titers, which were measured three days after inoculation did not show any difference in plant resistance between both tested groups (Additional file: 1 Figure S2A).

#### Nicotiana benthamiana

**Figure 3:**
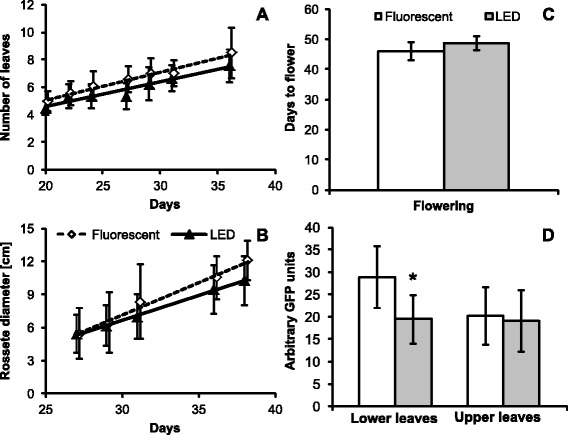
Growth of *N. benthamiana* plants under different illumination sources. Number of leaves **(A)** and rosette diameter **(B)** were recorded throughout the experiment. **C)** Average number of days from germination to the appearance of first flower. Values in panels A to C are based on one of two biological replicates, each group consisted of 11 plants. **D)** Total protein extracts from leaves inoculated with virus vector expressing GFP. One of two biological replicates, n = 9. Error bars represent SD.

Plant growth was measured as the diameter of emerging leaves at the beginning of the experiment (Figure 3B) and total plant length in later phases of the experiment. Number of leaves per plant was recorded throughout the experiment (Figure 3A), whereas other characteristics were recorded once per experiment: the appearance of first flowers; the weight of above ground plant biomass after 38 days; and flowering time (Figure 3C). Overall plants grown under both illumination sources showed very similar characteristics, with the LED grown plants being slightly slower both in appearance of new leaves and in flowering. Photosynthetic pigments were extracted and analyzed from 30 days old plants. Plants grown under LED illumination showed significantly elevated levels of neoxanthin, violaxanthin and antheraxanthin and b-carotene was decreased under LED (Additional file: 2 Figure S3B).

Thirty days after germination the plants were agroinfiltrated with Agrobacterium carrying a TMV-based viral vector expressing GFP. The fluorescence of leaf extracts containing the expressed GFP was measured on a fluorometer. Both experimental groups reacted similarly to agroinfiltration of the plant virus vector, with the first fluorescent foci appearing within 5 days upon infiltration. The rate of spread of viral infection was similar for both groups (data not shown). However, the plants grown under fluorescent tubes showed greater variation between older and younger leaves (Figure 3D). In fact, the age of the leaves did not have any impact on GFP expression when the plants were grown under LED illumination, while the lower (older) leaves of plants grown under fluorescent lamps showed significantly higher levels of GFP accumulation. The more balanced GFP levels might give an important advantage in expression experiments because it might help to reduce experimental variability and artefacts.

#### Glycine max

**Figure 4:**
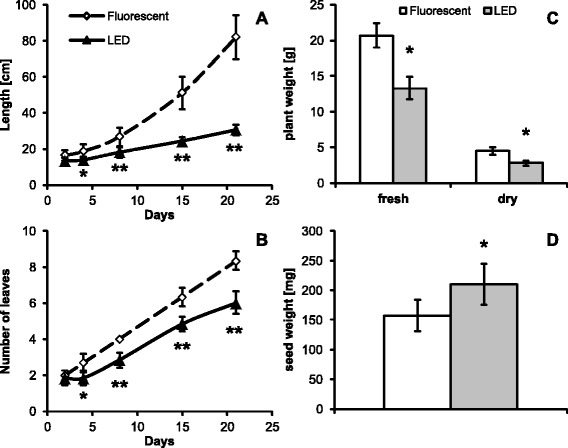
Growth of *G. max* plants under different illumination sources. Length **(A)** and number of trifoliate leaves **(B)** of the plants during the first month after replanting to 14x14 cm pots. **C)** Fresh and dry plant weight of above soil plant biomass. **D)** Individual mature seed weight. Curves and bars are based on data from one of two biological replications, 10 plants were used for treatment. Error bars represent SD.

From all the plant species tested, the largest photomorphogenic impact of the light source used was observed in soybean. Plants grown under fluorescent lights showed very rapid growth with an increasing internodal length (from 3 cm up to 20 cm, Figure 4A). By contrast, internodes of plants grown under the LED tubes were almost all of the same length of about 4.5 cm (Figure 4A). LED grown plants were also somewhat slower in developing new leaves (Figure 4B) (3 days) and in the appearance of first flowers (32 vs. 37 days after germination, Additional file: 3 Figure S4A; Additional file: 3 S4B). This difference was reflected also in the lower biomass harvested one month after germination (Figure 4C) and interestingly by a longer seed filling stage. This was reflected in a significantly higher weight of individual seeds (Figure 4D) in both biological replications of the experiment. The number of seeds per plant was significantly lower under LEDs in one of the biological replicates (54 vs. 25); however, this difference was insignificant in the second biological replication (48 vs. 45). Analysis of photosynthetic pigments showed increased levels of antheraxantin and violaxanthin and reduced levels of lutein, zeaxanthin and both chlorophylls in LED grown plants (Additional file: 3 Figure S4C).

#### Solanum tuberosum

**Figure 5:**
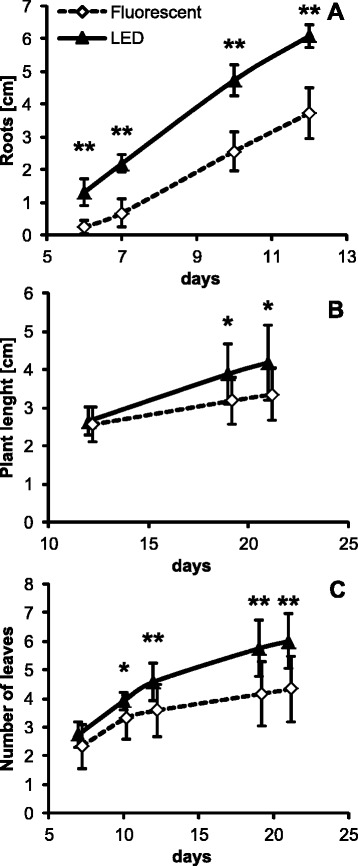
*In vitro* cultivation of *S. tuberosum* plants under different illumination sources: **A)** Average length of the longest root during the first two weeks after replanting of plant shoots to magenta boxes. The roots became too dense for further measurement after this period. **B)** Total shoot length. **C)** Average number of leaves per plant. **A-C** Plots are based on data from one of two biological replicates. 12 plants were used per treatment. Error bars represent SD. Asterisks indicate statistically significant differences compared to plants grown under fluorescent lights (*P < 0.05; **P < 0.01, two tailed Student’s *t*-test).

Potato explants were the only *in vitro* plants tested in this study. The rate of both root and shoot formation (Figure 5A) and their growth (Additional file: 4 Figure S5) was significantly higher under LED lights. Also, new leaves appeared faster in LED grown plants (Figure 5B). Plants under LED lights exhibited more leaves than under fluorescent tubes (Figure 5C). Plants under both light sources slowed down their growth and eventually reached a plateau phase after approximately 18 days when the shoots filled the Magenta boxes. The better growth under LED lights was also reflected in higher fresh and dry biomass (data not shown).

#### Brassica napus

**Figure 6:**
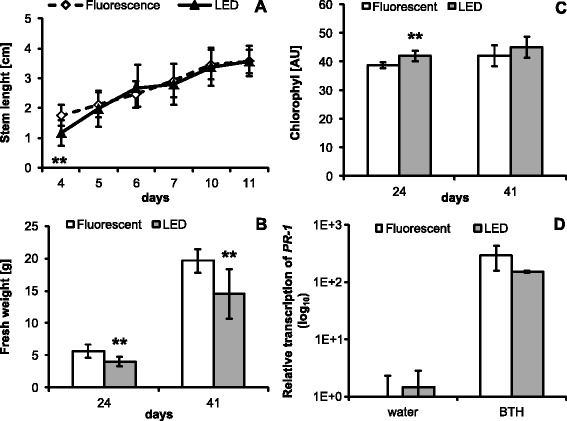
Growth of *B. napus* plants under different illumination sources. A) Stem length (n = 24). **B)** Fresh weight of whole 24 and 41 days old plants; **C)** chlorophyl content of the same plants (n = 15). **D)** Relative transcription of *PR1* gene (n = 3). The *PR1* transcription was normalized to the reference gene for actin. In all cases error bars represent SD. Asterisks indicate statistically significant differences compared to plants grown under fluorescent lights (**P < 0.01, two tailed Student’s *t*-test).

The growth of brassica seedlings was measured as stem length up to 11 days after germination (Figure 6A). Interestingly, the stem elongation of plants grown under LED lights was one day delayed compared with the plants under fluorescent light (Figure 6A and Additional file: 5 Figure S6A). After the seeds germinated the growth rate had similar dynamics in both groups and by the 11^th^ day size was similar for both variants (Figure 6A). We have further measured the fresh weight of whole plants on the 24^th^ and 41^st^ day. The plants grown under LED lights had lower biomass weight than plants grown under fluorescent lights (Figure 6B), which correlates with the higher number of true leaves for plants under fluorescent light (Additional file: 5 Figure S6B). These experiments indicate that LED lights delayed the development and aging *of Brassica napus* plants. Chlorophyll content was measured using a leaf clip device and on the 24^th^ day the LED grown plants showed statistically higher chlorophyll content but became insignificant on the 41^st^ day (Figure 6C). Similarly to the experiments with *Arabidopsis thaliana*, we also wanted to test the impact of illumination source on the transcription of the defense gene *PR1*. In this case we treated the plants with BION® (contains BTH – benzothiadiazole as the active ingredient) which is a commercially-available inducer of plant disease resistance [24]. BTH is the functional analog of SA which induces the transcription of defense genes, among others also *PR1*. No significant differences between the plants grown under the tested light sources were observed (Figure 6D).

## Discussion

Objective of this study was to examine the feasibility of using polychromatic LED tube lighting - in terms of providing sufficient light intensity and quality for plant growth and development in experimental growth chambers - and their potential to replace existing conventional fluorescent tubes. Important aspect of our effort was the overall economy of LED based solution and the requirement to limit the initial investment to minimum. While there are far superior LED arrays specifically designed for plant growth, they tend to be also rather more expensive. We have selected several plant species currently used in our laboratories and compared their growth under LED tubes with their growth under fluorescent lighting. In addition to basic plant growth, we have also performed several basic experiments aimed at assessing the plants’ response to stress.

In contrast to other LED-based plant growth systems which usually contain a mixture of chips emitting in narrow bands, the LED tubes used in this study provided a full and continuous visible spectrum with pronounced blue and red irradiation. The LED tubes we used are equipped with a standard G13 light fitting, thus they can be used directly in the existing infrastructure designed for conventional fluorescent tubes and do not require any potentially expensive reconstruction and electrical refitting. If desired, the tubes can even be mixed with standard fluorescent tubes. Also, since these standard LED tubes are intended for the mass consumer market, they can be purchased relatively inexpensively and future reductions in their price is to be expected. Tubes used in this work were borrowed from their manufacturer Frontier Technologies (Prague, Czech Republic) for the duration of the experiments.

Our work was motivated by efforts to reduce the costs related to energy consumption of the plant growth facilities at our institute; in this context the capacity of LED technology to reduce both energy requirements and heat generation could not be ignored.

The usefulness of light for plant growth and development is defined by its quality (spectral composition), quantity (photon flux) and duration of illumination (photoperiod). Light sources used in this work differed only in their spectral composition, while the photoperiod and quantity of light was kept either identical or closely similar (Figure 1B,C).

The photon flux measured by the Li-Cor Quantum Photometer showed almost identical values for both light sources.

### Light quality

With the fast progress in the development of the LED technology and especially considering its flexibility and low power consumption it is clear that this technology will be more and more used for indoor plant growth.

Fluorescent lamps are currently the most common source of light for indoor cultivation. However, they emit light in several narrow bands ranging from 350 to 750 nm and these are not always aligned with the wavelengths absorbed by a plant’s photosynthetic apparatus; they thus generate unnecessary heat. By contrast, the LEDs used in this work provide a continuous spectrum of all wavelengths between 400–700 nm, with enhanced radiation at around 450 nm and 665 nm. Contrary to conventional fluorescent tubes which are used as universal light source, the LEDs can be fine tuned for specific purpose (eg optimized for particular plant species, induction of flowering, change of morphology). Since it has been shown many times that light of various wavelengths acts not only as the energy source for photosynthesis but also as an effective growth regulator [2,21,25], we wanted to see whether two light sources with principally different spectral qualities could both be used in growth chambers to grow healthy experimental plants and what would be the impact of different light spectra on various physiological experiments. In some settings it might be important to compare the older experimental data gained using fluorescent tubes with newer datasets obtained from plants grown under LED illumination.

In one of the first studies of LED illumination being used for plant growth, Bula et al. (1991) used LEDs supplemented with blue fluorescent (BF) lamps and the effect on the lettuce plants studied was equivalent to that of cool-white fluorescent (CWF) lighting plus incandescent lamps [4]. However, Hoenecke et al. (1992) showed that plants grown only under LEDs which emitted mostly red light (660 nm) have different growth of hypocotyls and cotyledons. These effects were prevented by the addition of at least 15 μmol^.^m^-2.^s^−1^ of blue light [5]. This early work demonstrated that complex light sources are needed.

In previous work, Cope and Bugbee (2013) have also used continuous-spectrum LED-diodes and have shown that for some plant species the relative ratio of blue to red light is important while for some others the absolute amount of blue light is a better descriptor [21]. It has also been shown many times that green light opposes the effects of the red and blue wavebands (for an excellent review see [2]). As already mentioned in the results, the two used light sources differed mostly in their red component: this contributed almost 61% of total photon flux from LED tubes, while only up to 39% of photons from fluorescent lights.

#### Economy of use

The most important motivation for replacing conventional fluorescent tubes with LEDs is their lower power consumption, which also brings a substantial reduction in the heat generated and a reduction in water use. From our measurements it is clear that the LED-based solution provides an equivalent PPFD (photosynthetic photon flux density) while using only 38% of the energy consumed by fluorescent tubes. Their energy efficiency is mostly helped by the fact that all emitted photons are directed to a relatively narrow angle of 120° while the fluorescent tubes emit electrons in a full 360° circle. Additional power savings might be expected from the reduced need for air-conditioning; however, these savings are more difficult to estimate.

One interesting observation made during these experiments was that while the air-conditioning along with the passive airflow was adequate to keep the temperature quite near to the preset value in most of the growth room, there were spots of substantially higher temperatures on the growth shelves caused by the limited air flow between the plants and pots. When fluorescent lights were used the temperatures measured directly between the plants within the trays were 2.5° above the threshold, however the temperatures just few centimeters to the side reached 31.4°C creating quite steep temperature gradient. Such gradient was not observed when the LED lights were used. This was also reflected in increased water consumption of plants under fluorescent lights. While the high temperature spots could be efficiently controlled with a fan providing an active airflow, the absence of such gradient is an important advantage of the LED based solution which reduces the need for additional active elements in the growth chambers. We are fully aware that many if not all the differences in growth characteristics recorded throughout this work might be at least partially attributed to these differences in temperatures. The experimental design used in this study was designed to show differences in plant growth in the case when the fluorescent tubes would be replaced with the same number of LED light sources with otherwise unchanged cultivation settings. The lower air temperature resulting from the lower heat generation of LEDs is thus one of the principal findings of this study. Since we plan to use a larger number of LED tubes than the ones deployed in this study in the future, we also want to prepare an experimental design which will separate the effect of temperature from the spectral composition.

The reduced generation of heat by LED tubes was also reflected in the reduced consumption of water or nutrient solution by about one third (Figure 1D). This brings important savings in the time dedicated to watering and checking of plants. In our settings it has also reduced the water stress over weekends or longer holidays when plants under fluorescent light might have experienced overwatering combined with consequent drought, while plants under LEDs could be conveniently watered in longer (3-day) intervals.

Comparisons of the overall costs of LED tubes with the currently-deployed fluorescent tubes depends mostly on two factors – the initial investment and the cost of electricity [26]. Electric rates vary widely between countries and districts, thus the final decision as to whether the investment into converting to LED is profitable (and when) depends on a user’s geographical location. In our model, we have used current commercial rates in the Czech Republic of 2.5 CZK/kWh (approximately 0.11 USD), while the current price per tube is 1000 CZK (approximately 45.8 USD). Under this scenario the savings from reduced electricity consumption will balance the higher initial price of LED tubes after 25 months (16-hr daylight regime). We also expect that the prices of LED-based solutions will continue to decrease at a relatively fast rate, while electricity rates will continue to increase slowly, thus making the transition to LEDs even more attractive in the future.

Since our LED-based solution does not involve any rewiring of fixtures, we have assumed that the installation costs of both LED and fluorescent lamps to be the same; however, the fluorescent tubes would need to be replaced approximately 5 times during the lifetime of the LEDs, which would thus incur additional maintenance costs. Other costs related to periodic checking of the light output would be approximately the same irrespective of the type of lamp.

#### Growth and biomass

Of all the plant species tested, the largest difference in plant morphology was observed in soybean. In previous work Cope and Bugbee (2013) have shown the effect of blue light on the stem length of developing soybean plants. In their experiments the increasing absolute blue light of up to 50 μmol^.^m^-2.^s^−1^ resulted in decreased stem length. In our experiments both groups received a similar absolute amount of blue radiation (28–31 μmol^.^m-^2.^s^−1^ or 32–35 μmol^.^m^−2^_._s^−1^ for fluorescent and LED grown plants, respectively) and also similar were its relative proportions to other wavelengths (16.1% vs. 19.1%). Clearly the very fast growth rate of shoots in plants under fluorescent lights cannot be explained by the differences in blue light irradiation alone. It is true, however, that the amount of blue light in both groups was near the saturation point observed by Cope and Bugbee and thus other components might have played a role.

Another contributing factor might be that we have used cultivar Jack as opposed to the dwarf variety Hoyt. For the growth of experimental plants it is important that LED-grown plants are substantially more compact and thus better fit into the limited space of the growth chamber. On the other hand, both their flowering and seed filling was delayed, which is a drawback that needs to be taken into account when planning experiments. Since the LED tubes emit very little energy in the far-red region, it would be interesting to see if this delay could be reverted by some additional source of far-red illumination.

It is also interesting to note that out of all the measured photosynthetic pigments, the most striking difference between fluorescent- and LED-grown soybeans was in the reduced levels of zeaxanthin under LED illumination; zeaxanthin has a role in the dissipation of excess excitation energy by participating in non-photochemical quenching and is essential in protecting the chloroplast from photo-oxidative damage [27]. Thus the plants grown under fluorescent lights have exhibited very fast rates of elongation of shoots, which is a common reaction to insufficient light, and at the same time increased levels of pigments protecting them from photodamage.

Another striking difference observed during described set of experiments was the relative speed of root formation by potato explants *in vitro*. LED grown plants started to root practically immediately after placement into solid media, while under fluorescent light the first shoots started to appear after one week. It is very likely that the plants might have been stressed by high temperatures inside of the magenta box under fluorescent lights. The higher temperature in magenta also probably affected the water content in growing plants, thus the plants growing under LED contained more water and less dry matter than the plants grown under fluorescent light (data not shown).

Other plant species tested have shown very similar growth characteristics and biomass accumulation under both light sources, albeit sometimes slightly slower growth under LED lights, which again can be fully explained by the slightly decreased temperature.

#### Plant response to stress

It is known that plant immunity is modulated by both the quantity and quality of light and by temperature [28,29]. In this set of experiments we have observed the plant response to several stressors, namely in the canonical pathosystems *Arabidopsis thaliana* x *Pseudomonas syringae*, *N.benthamiana* x Tobacco mosaic virus, and *Brassica napus* x *Leptosphaeria maculans.* In the *Arabidopsis* system we did not observe any statistically significant differences in plant resistance to *Pseudomonas* (Additional file: 1 Figure S2A). It was shown previously that light has an effect on the salicylic acid (SA) signaling pathway [30]. We measured the transcription level of *PR1* (*PATHOGENESIS RELATED 1*) gene (marker gene of SA signaling) in both *Arabidopsis* and *Brassica napus*. We have shown that basal levels of *PR1* transcription were elevated in Arabidopsis plants under LED light (Figure 2D). This is in agreement with the observation of Wang et al. (2010), who showed that red light induces *PR-1* transcription in cucumber [31]. However, these elevated basal levels did not have any measurable effect on *Arabidopsis* resistance to *Pseudomonas*; similarly, for Brassica the increase was very little.

Agroinfiltrated *N. benthamiana* leaves of both groups also appeared almost identical under UV light. Interestingly, when the fluorescence of extracts was measured using a fluorometer, the LED grown plants showed a lower variation in accumulated GFP between older and younger leaves (Figure 3B), which might be an important advantage in the study of plant virus interactions. Based on these observations we believe that the LED light system is suitable for the study of plant-microbe interactions.

## Conclusions

Based on our study we propose that the polychromatic LED tubes are suitable for the indoor cultivation of multiple species of experimental plants destined for general plant research. The main advantages of LEDs are lower energy costs, lower heat generation, lower demands for watering, and the longer lifetime of the light source. Some plants, however, might grow slightly slower, thus reducing the advantage in power consumption.

It is not surprising that the responses of different plant species varied from very minor (e.g. *Arabidopsis*) to a considerable change in morphology (soybean) and/or speed of root formation and growth (*in vitro* cultivated potato plantlets).

From our point of view this study can serve as a fundamental source of data for plant scientists who are considering partial or full transition from fluorescent to solid state illumination sources.

## Materials and methods

### Growth room

A plan of the place of cultivation is shown in Additional file: 6 Figure S1A. The temperature in the growth rooms was set to 21°C throughout the experiment. All plants were grown in a 14/10 hr light/dark regimen. The growth room contained five stands with three shelves each. Shelves were 140 cm wide, 80 cm deep and the distance from the illumination source to the shelf surface was 43 cm. Each shelf was illuminated with either 8 standard 36 W fluorescent tubes (Philips TL-D Super 840) or with the same number of LED tubes with (T8 120 GrowLight, Frontier Technologies, Czech Republic). Each LED tube was fitted with 132 phosphor coated InGaN chips (Epistar SMD 2835). The stands were separated by white non-transparent barriers to prevent mixing of different light sources. Light intensity was measured using a Quantum Photometer LI-185B (Li-Cor, USA), equipped with a LI-190 quantum sensor calibrated for each treatment using the spectroradiometer data. The spectra of each respective light source were measured in situ using an Ocean Optics USB2000+ spectrometer and verified at the certified laboratory of the Technical University Ostrava using a JETI SPECBOS 1211 spectroradiometer. The blue/green/red light bands were defined as 400–500 nm, 501–599 nm, and 600–700 nm respectively [21]. The chlorophyll content of live plants was measured using a SPAD 502 meter (Minolta, Japan). Temperature was measured using a Silicon Labs USB Datalogger, which was either shielded or not from direct exposure to light. Datalogger was placed 7 cm above the shelf either on *Arabidopsis* leaves or not. Additional temperature measurements were made using a set of mercury thermometers submerged in a 250 ml Erlenmayer flask with 100 ml of distilled water and placed for at least two hours in various positions over the shelves to reach equilibrium.

### Photosynthetic pigments analysis

The content of photosynthetic pigments (Chl’s *a* and *b*, β-carotene, lutein, neoxanthin, violaxanthin, zeaxanthin and antheraxanthin) was determined in acetone extracts made from the lyophilized leaves by HPLC (ECOM, Czech Republic). The analysis was made using a reversed phase column (Watrex Nucleosil 120 5 C18, 5 μm particle size, 125 × 4 mm, ECOM, Czech Republic), the solvent system comprised of acetonitrile:methanol:water (80:12:10 v:v:v) followed by methanol/ethylacetate (95:5 v:v), the total analysis time was 25 min, and the linear gradient was run from 2 to 6 min (the flow rate 1 cm^3^ min^−1^, the detection wavelength 445 nm). Data were captured and calculated by PC-software Clarity (DataApex, Czech Republic).

#### Arabidopsis thaliana

Seeds of *Arabidopsis thaliana* ecotype Col-0 were vernalized 3 days at 4°C in soil, after which they were placed in a Snijders (microclima *Arabidopsis* cabinet model MCA 1600E-7TL) growth chamber where they were grown in soil at 22°C on a 10 h day (130 μmol.m^−2^.s^−1^) and 14 h night cycle at 70% relative humidity for one week. One week old plantlets were individually replanted to Jiffy 7 peat pellets and placed in the cultivation room under the experimental light conditions. During the duration of the experiment plants were watered with no fertilizer. For gene expression analyses four-five weeks old plants were sprayed with 0.3 mM sodium salicylate (Sigma-Aldrich) or with distilled water for controls. Leaves were collected 8 h after treatment and frozen in liquid nitrogen. For the bioassay with *Pseudomonas* plants were 5 weeks old.

#### Bacterial inoculation

*Pseudomonas syringae* pv. *maculicola* ES4326 were grown on King B agar plates at 28°C overnight, resuspended in 10 mmol MgCl_2_ and diluted to an OD600 of 0.5. Silwet L77 was added to the bacterial suspension to give a final concentration of 0.02% and plants were sprayed until runoff. Plants were enclosed in a transparent airtight container for 24 h to maintain a high relative humidity and were collected 3 dpi. Approximately 40 mg of 0.6-mm diameter leaf discs were homogenized in tubes with 1 g of 1.3 mm silica beads using a FastPrep-24 instrument (MP Biomedicals,CA, USA). The resulting homogenate was serially diluted and loaded onto King B plates. Colonies were counted after 2 d of incubation at 28°C.

#### Nicotiana benthamiana

Seeds were first germinated on a sand:soil 1:1 mixture in a high humidity chamber. The 18 to 21 days old plantlets were transferred to soil into larger pots (8x8 cm diameter). Plants were grown in a mixture consisting of neutral gardening substrate: sand: perlite 4:1:2 in these pots thorough the experiment. Immediately after repotting, the plants were moved to a growth room with either LED or fluorescent illumination.

*Virus inoculation:* Fully expanded leaves of *N. benthamiana* were agroinfilatrated, essentially as described in Cerovska et al., (2008) except for using TMV replicon expressing GFP (GenBank accession number KF981446) instead of PVX based vector [32]. On each plant three fully developed leaves were infiltrated with 200 ul of *Agrobacterium* suspension (OD600 = 1.0 in infiltration solution 10 mM MES, 200 mM acetosyringone, 10 mM MgCl_2_). Duplicate samples (1 cm) were collected from each plant under UV illumination to ensure processing of only virus-infected tissue. Leaf tissue was homogenized in 400 μl PBS buffer using ceramic beads and a FastPrep 24 instrument and total protein content measured using total protein assay (BioRad). Samples were then equilibrated to 1 mg/mL total protein concentration and GFP fluorescence measured using a Tecan-F200 instrument (Tecan, Austria).

#### Glycine max

Individual seeds were placed into Jiffy peat pellets and incubated at 28°C in dark and humid conditions for 48 hours. The plants were then transferred to the experimental growth room. 16 days after germination the plants were replanted to square pots (7x7 cm, 230 mL) containing a mixture of gardening substrate: sand: perlite (4:1:2). Shoot length, number of trifoliate leaves, and flowering time was recorded at 2–3 days intervals throughout the whole experiment. Finally, 45 days after germination, i.e. approximately one week after flowering, the plants were harvested and their fresh and dry weight recorded. Four plants per group were then replanted to larger pots (13x13 cm, 1.45 L) and left to reach maturity and harvest the seeds.

#### Solanum tuberosum

*In vitro* cultivated plantlets of potato cultivar Kamýk (breeder Selekta Pacov plc., Czech Republic) were grown under fluorescent illumination prior to the experiment. The tops of plantlets with three leaves were cut and placed on solid MS medium supplemented with 2% of sucrose in magenta boxes (4 plants per box). Then the boxes were transferred to the experimental growth room, 16 plants each under either LED or fluorescent illumination. Root growth, shoot length, and number of leaves per plant were recorded during the experiment and finally the fresh weight and dry weight of plants was recorded.

#### Brassica napus

Plants *Brassica napus* cv. Columbus were grown hydroponically in perlite in Steiner’s cultivation medium 9 (Steiner, 1984). The plants were in trays each containing four sextuplet pots. Cotyledons of 11-day-old plants were used for 30 uM benzithiadiazole (BTH, BION®) treatment by spraying.

### Gene expression analysis

Leaves from 4–5 weeks old plants (≈150 mg) were collected for each sample and were immediately frozen in liquid nitrogen. The tissue was homogenised in tubes with 1 g of 1.3 mm silica beads using a FastPrep-24 instrument (MP Biomedicals,CA, USA). Total RNA was isolated using a Spectrum Plant Total RNA Kit (Sigma-Aldrich) and treated with a DNA-free Kit (Ambion, Austin, TX, U.S.A.). Subsequently, 1 μg of RNA was converted into cDNA with M-MLV RNase H– Point Mutant reverse transcriptase (Promega Corp.) and anchored oligo dT21 (Metabion, Martinsried, Germany). An equivalent of 6.25 ng of RNA was used as template in 10-μl reaction with a qPCR mastermix EvaLine – E1LC (GeneOn, Ludwigshafen am Rhein, Germany). All reactions were performed in polycarbonate capillaries (Genaxxon, Ulm, Germany) on LightCycler 1.5 (Roche). The following PCR program was used for all PCR assays: 95°C for 10 min; followed by 45 cycles of 95°C for 10 s, 55°C for 10 s, and 72°C for 10 s; followed by a melting curve analysis. Threshold cycles and melting curves were calculated using LightCycler Software 4.1 (Roche). Relative expression was calculated with efficiency correction and normalization to *SAND*. Primers were designed using PerlPrimer v1.1.17 (Marshall 2004). The following is the list of *A. thaliana* genes and corresponding accession numbers and primers: *SAND*, AT2G28390, FP: 5′CTG TCT TCT CAT CTC TTG TC 3′, RP: 5′ TCT TGC AAT ATG GTT CCT G 3′, *PR-1*, AT2G14610, FP: 5′ AGT TGT TTG GAG AAA GTC AG 3′, RP: 5′ GTT CAC ATA ATT CCC ACG A. The following is the list of *B. napus* genes and corresponding accession numbers and primers: *Actin*, AF111812, FP: 5′-CTG GAA TTG CTG ACC GTA TGA G-3′, RP: 5′-TGT TGG AAA GTG CTG AGG GA-3, *PR-1*, BNU21849, FP: 5′-CAT CCC TCG AAA GCT CAA GAC-3′, RP: 5′-CCA CTG CAC GGG ACC TAC-3′.

## Additional files

Additional file 1: Figure S2.
*Arabidopsis thaliana* A) *Pseudomonas syringae* pv maculicola ES4326 titres in the leaves collected at 0 and 3 days post infection (n=5). B) Representation of dry matter in plants (n=11; 25 and 42 days) and (n=6; 36 days). C) Representative image of 8 weeks old plants. D) Photo of the same age plants. E) Image of rosettes of 4-week old plants which were used for weight measurement. Error bars represent SD. Statistically significant differences compared Fluorescent vs LED (*P<0.05; Student’s t-test). Additional file 2: Figure S3.
*Nicotiana bentamiana*. **A)** Plant growth and development under studied ilumination sources. **B)** Relative contents of photosythetic pigments determined by HPLCA chromatography. Values obtained from plants under fluorescent light are 100%Five leaves per treatment were analyzed, error bars represent SD, Statistically significant differencescompared fluorescent vs LED light conditions (*P<0.05; **P<0.01, Student’s t-test). Additional file 3: Figure S4.
*Glycine max*. **A)** Growth of the plants. **B)** Days from germination to appearance of the firstf lowers. Plants grown under fluorescent lights produced significantly longer internodes and shorter vegetative period. **C)** Relative contents of photosynthetic pigments determined by HPLC chromatography. Values obtained from plants under fluorescent light are 100%. Five leaves per treatment were analyzed. Error bars represent SD. Statistically significant differences compared fluorescent vs LED (*P<0.05;**P<0.01, Student’s t-test). Additional file 4: Figure S5.
*Solanum tuberosum* **.** Emerging roots seven days after replanting. LED-grown plants posses significantly longer roots. Additional file 5: Figure S6.
*Brassica napus*. **A)** Photo of plants shortly after germination. **B)** Number of leaves from 15 plants. Error bars represent SD. Statisticaly significant differences compared fluorescent vs LED light conditions(*P<0.05; **P<0.01; Student’s t-test). Additional file 6: Figure S1. Shape and dimensions (in cm) of cultivation frame.
