# Supplementary material for: Growth and stress response in Arabidopsis thaliana, Nicotiana benthamiana, Glycine max, Solanum tuberosum and Brassica napus cultivated under polychromatic LEDs
Source: Plant Methods. 2015 Apr 30;11:31. doi: 10.1186/s13007-015-0076-4 (PMC4940826; doi:10.1186/s13007-015-0076-4)
Supplement: Additional file 1: Figure S2. — Arabidopsis thaliana A) Pseudomonas syringae pv maculicola ES4326 titres in the leaves collected at 0 and 3 days post infection (n=5). B) Representation of dry matter in plants (n=11; 25 and 42 days) and (n=6; 36 days). C) Representative image of 8 weeks old plants. D) Photo of the same age plants. E) Image of rosettes of 4-week old plants which were used for weight measurement. Error bars represent SD. Statistically significant differences compared Fluorescent vs LED (*P<0.05; Student’s t-test). [file 13007_2015_76_MOESM1_ESM.pdf]

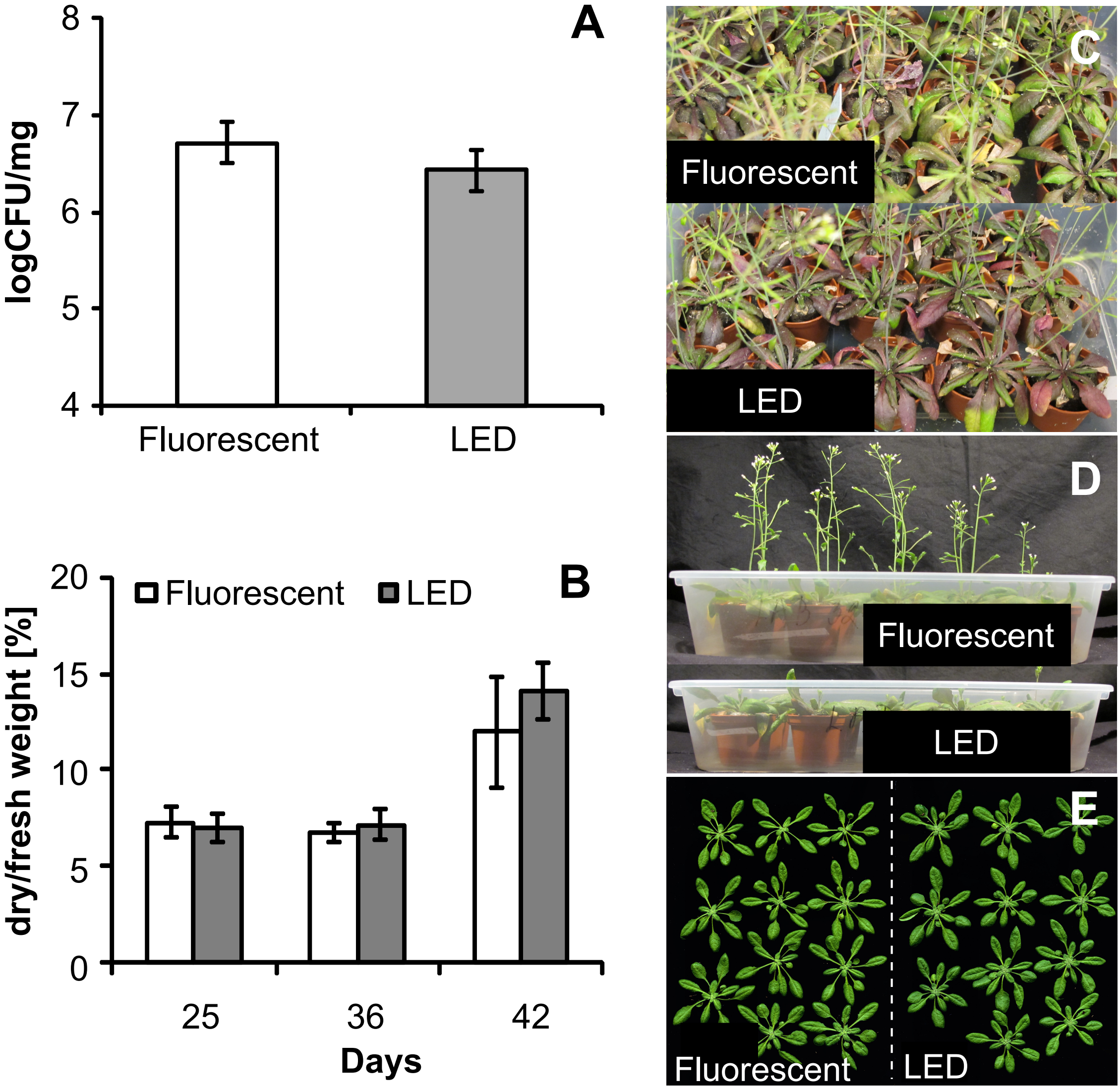

Figure S2 ***Arabidopsis thaliana*** **A)** *Pseudomonas syringae* pv. *maculicola* ES4326 titres in the leaves collected at 0 and 3 days post infection (n=5). **B)** Representation of dry matter in plants (n=11; 25 and 42 days) and (n=6; 36 days). **C)** Representative image of 8 weeks old plants. **D)** Photo of the same age plants. **E)** Image of rosettes of 4-week old plants which were used for weight measurement. Error bars represent SD. Statistically significant differences compared fluorescent vs LED (*P<0.05; Student´s t-test)
