# Supplementary material for: Growth and stress response in Arabidopsis thaliana, Nicotiana benthamiana, Glycine max, Solanum tuberosum and Brassica napus cultivated under polychromatic LEDs
Source: Plant Methods. 2015 Apr 30;11:31. doi: 10.1186/s13007-015-0076-4 (PMC4940826; doi:10.1186/s13007-015-0076-4)
Supplement: Additional file 2: Figure S3. — Nicotiana bentamiana. A) Plant growth and development under studied ilumination sources. B) Relative contents of photosythetic pigments determined by HPLCA chromatography. Values obtained from plants under fluorescent light are 100%Five leaves per treatment were analyzed, error bars represent SD, Statistically significant differencescompared fluorescent vs LED light conditions (*P<0.05; **P<0.01, Student’s t-test). [file 13007_2015_76_MOESM2_ESM.pdf]

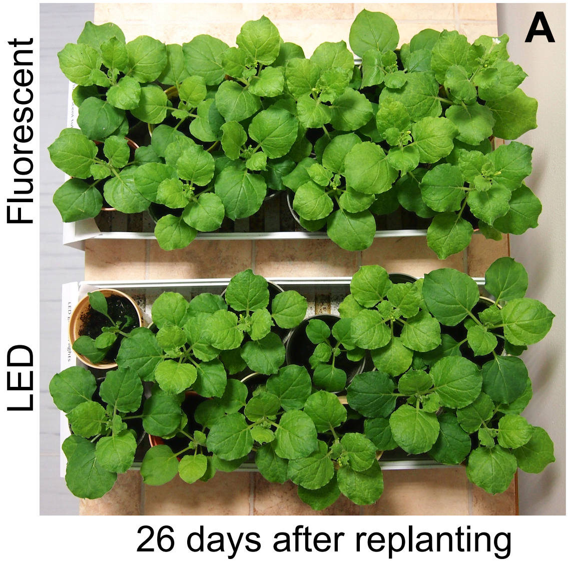

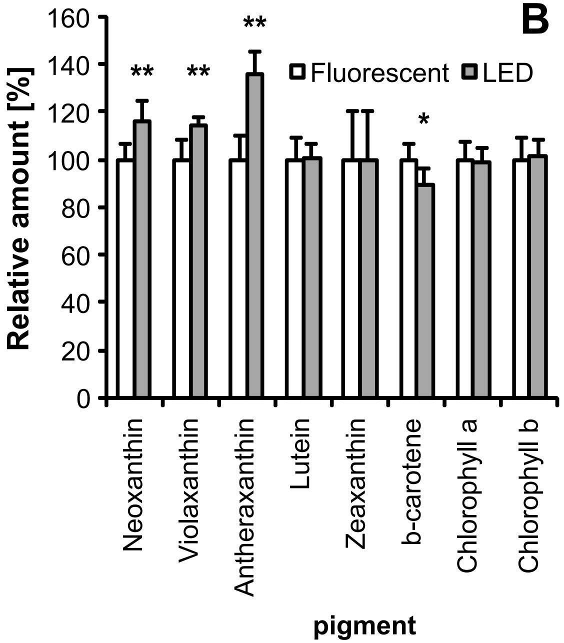

Figure S3. Nicotiana bentamiana. A) Plant growth and development under studied ilumination sources. **B)** Relative contents fo photosythetic pigments determined by HPLC chromatography. Values obtained from plants under fluorescent light are 100 %. Five leaves per treatment were analyzed, error bars represent SD. Statistically significant differences compared fluorescent vs LED light conditions (*P<0.05; **P<0.01, Student´s t-test).
