# Supplementary material for: Growth and stress response in Arabidopsis thaliana, Nicotiana benthamiana, Glycine max, Solanum tuberosum and Brassica napus cultivated under polychromatic LEDs
Source: Plant Methods. 2015 Apr 30;11:31. doi: 10.1186/s13007-015-0076-4 (PMC4940826; doi:10.1186/s13007-015-0076-4)
Supplement: Additional file 3: Figure S4. — Glycine max. A) Growth of the plants. B) Days from germination to appearance of the firstf lowers. Plants grown under fluorescent lights produced significantly longer internodes and shorter vegetative period. C) Relative contents of photosynthetic pigments determined by HPLC chromatography. Values obtained from plants under fluorescent light are 100%. Five leaves per treatment were analyzed. Error bars represent SD. Statistically significant differences compared fluorescent vs LED (*P<0.05;**P<0.01, Student’s t-test). [file 13007_2015_76_MOESM3_ESM.pdf]

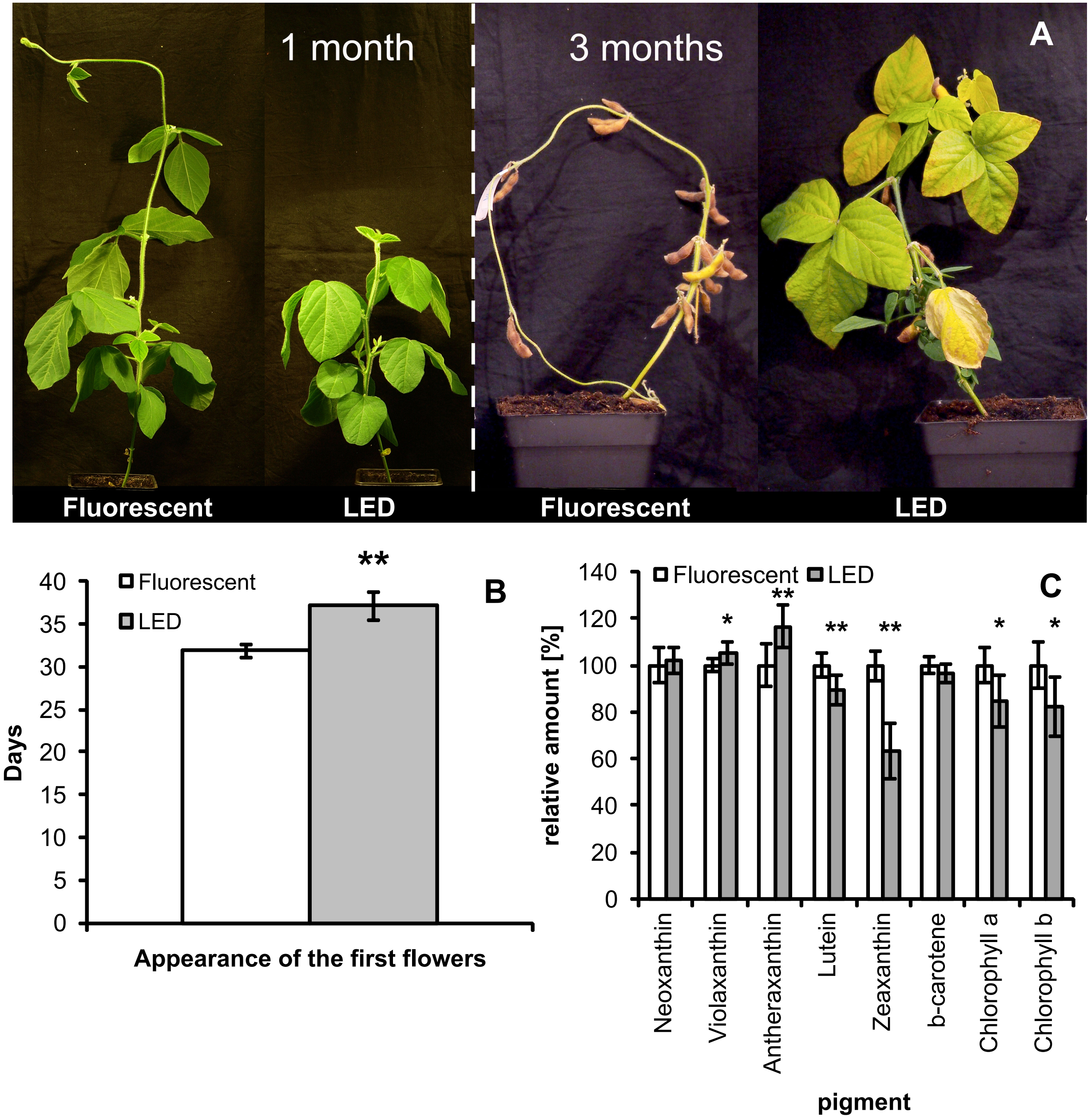

Figure S4. ***Glycine max.*** **A)** Growth of the plants. **B)** Days from germination to appearance of the first flowers. Plants grown under fluorescent lights produced significantly longer internodes and shorter vegetative period. **C)** Relative contents of photosynthetic pigments determined by HPLC chromatography. Values obtained from plants under fluorescent light are 100 %. Five leaves per treatment were analyzed, error bars represent SD. Statistically significant differences compared fluorescent vs LED (*P<0.05; **P<0.01; Student´s t-test)
