# Supplementary material for: Growth and stress response in Arabidopsis thaliana, Nicotiana benthamiana, Glycine max, Solanum tuberosum and Brassica napus cultivated under polychromatic LEDs
Source: Plant Methods. 2015 Apr 30;11:31. doi: 10.1186/s13007-015-0076-4 (PMC4940826; doi:10.1186/s13007-015-0076-4)
Supplement: Additional file 4: Figure S5. — Solanum tuberosum . Emerging roots seven days after replanting. LED-grown plants posses significantly longer roots. [file 13007_2015_76_MOESM4_ESM.pdf]

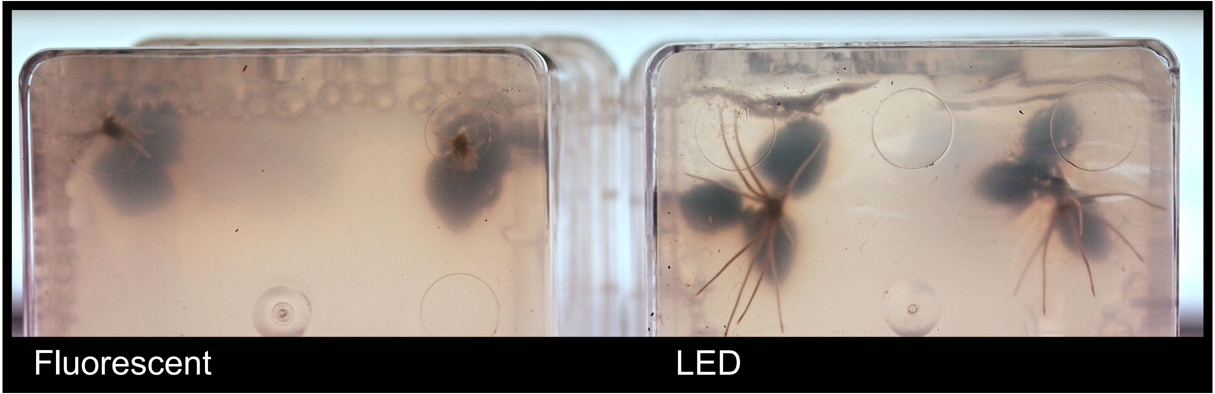

Figure S5. ***Solanum tuberosum*****.** Emerging roots seven days after replanting. LED-grown plants posses significantly longer roots.
