# Supplementary material for: Growth and stress response in Arabidopsis thaliana, Nicotiana benthamiana, Glycine max, Solanum tuberosum and Brassica napus cultivated under polychromatic LEDs
Source: Plant Methods. 2015 Apr 30;11:31. doi: 10.1186/s13007-015-0076-4 (PMC4940826; doi:10.1186/s13007-015-0076-4)
Supplement: Additional file 5: Figure S6. — Brassica napus. A) Photo of plants shortly after germination. B) Number of leaves from 15 plants. Error bars represent SD. Statisticaly significant differences compared fluorescent vs LED light conditions(*P<0.05; **P<0.01; Student’s t-test). [file 13007_2015_76_MOESM5_ESM.pdf]

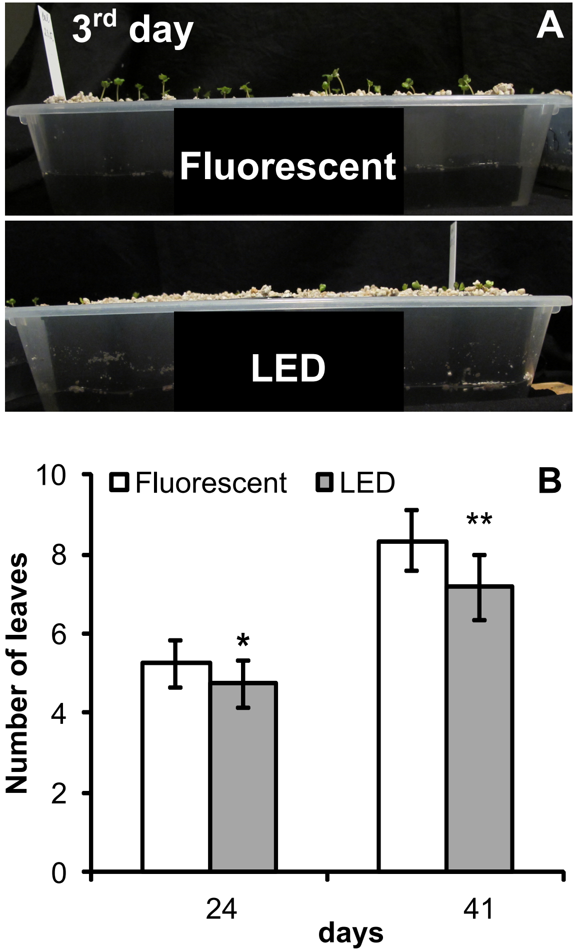

Figure S6. *Brassica napus*. A) Photo of plants shortly after germination. **B)** Number of leaves from 15 plants. Error bars represent SD. Statisticaly significant differences compared fluorescent vs LED light conditions (*P<0.05; **P<0.01; Student´s t-test).
