# Supplementary figures and images for: Growth and stress response in Arabidopsis thaliana, Nicotiana benthamiana, Glycine max, Solanum tuberosum and Brassica napus cultivated under polychromatic LEDs
Source: Plant Methods. 2015 Apr 30;11:31. doi: 10.1186/s13007-015-0076-4 (PMC4940826; doi:10.1186/s13007-015-0076-4)

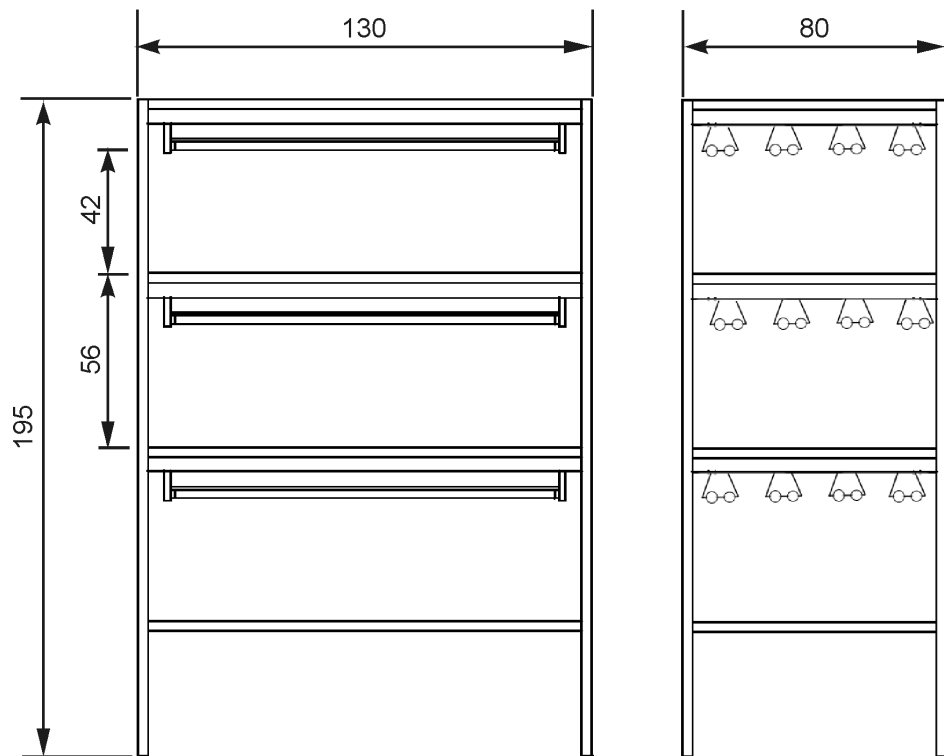

**Figure S1** Shape and dimensions (in cm) of cultivation frame.

Supplement: Additional file 6: Figure S1. — Shape and dimensions (in cm) of cultivation frame. [file 13007_2015_76_MOESM6_ESM.pdf]
